# Seeing the good in the bad: actual clinical outcome of thrombectomy stroke patients with formally unfavorable outcome

**DOI:** 10.1007/s00234-022-02920-1

**Published:** 2022-03-08

**Authors:** Friederike Blum, Charlotte Hager, Homan Taufik, Martin Wiesmann, Dimah Hasan, Arno Reich, João Pinho, Omid Nikoubashman

**Affiliations:** 1 Department of Neuroradiology, University Hospital RWTH Aachen, Pauwelsstr. 30, 52074 Aachen, Germany; 2 Department of Neurology, University Hospital RWTH Aachen, Pauwelsstr. 30, 52074 Aachen, Germany

**Keywords:** Ischemic stroke, Thrombectomy, Reperfusion, Outcome research, Interventional neuroradiology

## Abstract

**Purpose:**

Clinical outcome of stroke patients is usually classified into favorable (modified Rankin scale (mRS) 0–2) and unfavorable (mRS 3–5) outcome according to the modified Rankin scale. We took a closer look at the clinical course of thrombectomy stroke patients with formal unfavorable outcome and assessed whether we could achieve our treatment goals and/or neurological improvement in these patients.

**Methods:**

We studied 107 patients with occlusions in the terminal carotid artery or the M1 segment of the middle cerebral artery, in whom complete recanalization (eTICI 3) could be achieved, and who had an mRS of 3–5 at 90 days. We analyzed whether an individual treatment goal (i.e., preventing aphasia) and neurological improvement (NIHSS) could be achieved. In addition, we examined whether there was clinical improvement on the mRS.

**Results:**

The treatment goal was achieved in 52% (53/103) and neurological improvement in 65% (67/103). mRS 90 days post-stroke was better than mRS upon admission in 36% (38/107) and better than or equal to mRS upon admission in 80% (86/107). Of the 93 patients with known pre-stroke mRS, 18% (17/93) already had an mRS ≥ 3, with 15 of these 17 patients having a worse mRS on admission than before. Of these 17 patients, 18% regained baseline, and 24% improved from admission.

**Conclusion:**

Dichotomizing the mRS into favorable and unfavorable outcome does not do justice to the full spectrum of stroke. Patients with formal unfavorable outcome after mRS can improve neurologically, achieve treatment goals, and even regain their admission or pre-stroke mRS.

**Supplementary Information:**

The online version contains supplementary material available at 10.1007/s00234-022-02920-1.

## Introduction

Endovascular therapy is a standard procedure for large vessel occlusion stroke [1]. The modified Rankin scale score (mRS score), which describes stroke-related disabilities, is the most common parameter to assess the outcome of stroke patients [2–4]. Other common measures are the Barthel index as a measure of activities of daily living or the functional independence measure (FIM), which is mainly used to describe rehabilitation [5–8].

In most of the current literature related to stroke outcome, namely in many of the randomized controlled trials for acute phase treatments, a mRS 0–2 is considered a favorable outcome with functional independence [9–11]. This means that the patient lives independently without being dependent on external help or aids [12]. Such a favorable outcome (mRS 0–2) is achieved in about 30–70% of stroke patients with emergent large vessel occlusion (ELVO), depending on the study inclusion criteria [9, 10, 13–16]. Vice versa, this means that stroke outcome is unfavorable in a large portion of patients. For trials trying to demonstrate the benefit of an intervention, the dichotomization of mRS ensures that the primary outcome is robust and that the statistical analyses using this outcome are simple and reliable [17]. The trials which demonstrated the benefit of endovascular treatment of ELVO did not include patients with relevant pre-stroke disability [10, 15]. However, in a real-life setting, many patients present pre-stroke functional dependence, which introduces further complexity when trying to study the benefit of a therapeutic intervention in this setting. Even though the mRS score is supposed to capture stroke-related disabilities, it is often difficult to differentiate between actual stroke-related disabilities and pre-existing or aggravating conditions in these patients [18]. Stroke therapy may be beneficial in previously dependent patients, but the definition of “benefit” must take into consideration pre-stroke functional status and expected treatment goals. For example, the target of stroke therapy could be prevention of aphasia, even though hemiparesis is inevitable because there is already an established infarction involving the basal ganglia or motor cortex. Thus, patients with a seemingly unfavorable outcome according to mRS may have had the best outcome possible.

The aim of our study was to have a closer look into the clinical course of thrombectomy stroke patients with good procedural but still a formally unfavorable outcome (mRS 3–5) and to assess whether we achieved our treatment goals and/or neurological improvement in these patients.

## Methods

### Patients and methods

Data from the stroke registry of BLINDED were used for this analysis. This study was approved by the local ethics committee (approval reference BLINDED).

Our hospital is a tertiary stroke center with a catchment area of approximately 1 million inhabitants. We provide stroke therapy 7 days a week and 24 h a day. Available acute revascularization therapies include intravenous thrombolysis, mechanical thrombectomy, and carotid surgery [19]. If patients arrive at the hospital within a 4.5-h window after symptom onset or if there is relevant tissue at risk beyond this time window, intravenous thrombolysis is administered unless contraindications exist [20]. If the patient has a large-vessel occlusion, thrombectomy is performed regardless of the time window, provided that computed tomography (CT) including perfusion imaging implies that there is clinically relevant tissue at risk [21, 22].

For this analysis, we included all patients between May 2010 and July 2020 who met the following inclusion criteria: consecutive thrombectomy patients with occlusions in the terminal carotid artery or the M1 segment of the middle cerebral artery (with and without involvement of the bifurcation) in whom complete recanalization (expanded Thrombolysis in Cerebral Infarction; eTICI 3) was achieved and who had an mRS score of 3–5 at 90 days. We only included patients in whom we achieved complete recanalization, because reperfusion of an affected area is an important prerequisite for its recovery [23].

For these patients, several variables were prospectively collected: the mRS score pre-stroke, at admission and at dismissal, and after 90 days. Further, the National Institutes of Health Stroke Scale (NIHSS) on admission and at dismissal, and the Alberta Stroke Program Early CT Score (ASPECTS) initial and final.

Our two primary outcome parameters were (1) neurological improvement, defined as any neurological improvement on the NIHS scale at dismissal compared to neurological status upon admission and (2) achievement of a treatment goal, defined as the achievement of an individually predefined treatment goal.

Treatment goals were classified as (1) prevention of aphasia, (2) prevention of neglect, (3) prevention of paresis, or (4) a combination of two or three symptoms. To identify treatment goals, two independent experienced investigators (DH, a senior neuroradiologist, and JP, a senior neurologist) retrospectively reviewed clinical and radiological records and defined goals. Disagreements between the two investigators were settled, and a reference standard for statistical analyses was obtained in a consensus reading. The investigators classified the goals based on the specific records, first and foremost on the specifically written indications such as “thrombectomy for treatment of aphasia.” If no specific indications were stated, the goal was defined retrospectively according to baseline clinical and radiological characteristics. In patients with neurological deficits in specific eloquent regions (e.g., aphasia and motor and sensory speech regions; neglect and right hemispheric cortical regions; hemiparesis and internal capsule/corona radiata/frontal cortex) and without signs of infarction in the respective areas on noncontrast CT, a treatment goal for improvement of these functions was defined. [21, 22] For example, the treatment goal was defined as “treatment of aphasia” in a patient with right-sided hemiparesis and global aphasia who had infarction on noncontrast CT in the left precentral gyrus, but not in the centers for motor or sensory speech.

We also investigated clinical improvement on the mRS scale. Primary favorable outcome measure was defined as reaching pre-stroke baseline mRS; secondary favorable outcome measure was any neurological improvement after 90 days compared to admission.

### Statistics

After testing data distribution with a Shapiro–Wilk test, we applied Mann–Whitney *U* tests for data comparison. Nonparametric variables are indicated as median with interquartile range (IQR). Multivariable analysis was performed with a binary logistic regression test using the backward method (likelihood ratio), indicating odds ratios (ORs) and 95% confidence intervals (CI). Variables for regression analysis were selected in a hypothesis-driven manner. We examined the influence of age, sex, NIHSS at admission, initial ASPECTS on our results, as these are variables that can be collected reliably and objectified well. *p* values under the α-level of 0.05 were defined as significant. All statistical analyses were performed with SPSS software version 26 (IBM, Armonk, NY).

## Results

**Fig. 1:**
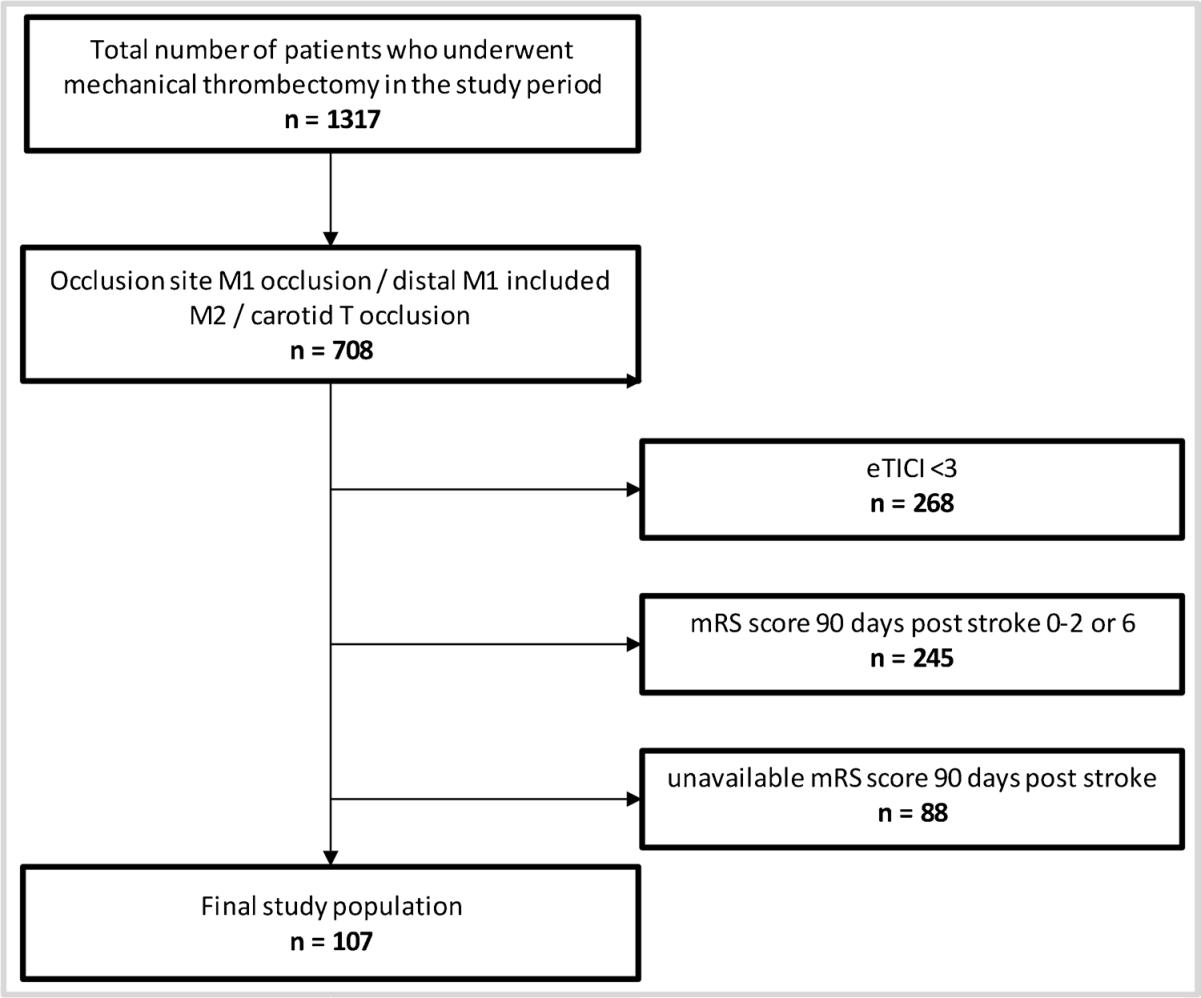
Patient flow diagram. mRS modified Rankin Scale, eTICI expanded thrombolysis in cerebral infarction

According to our inclusion criteria, 107 patients could be included in our study (Fig. 1). In 103 of these patients, we were able to collect a NIHSS and a treatment goal. Baseline, procedural, and outcome parameters of all patients can be found in the online supplemental data. The median age of all 107 patients was 77 years (IQR, 71–83). There were 64 female patients (60%). Median NIHSS upon admission was 18 (IQR, 12–20). Median pre-stroke mRS score was 0 (IQR, 0–1) in 93 patients, in whom pre-stroke mRS was assessable. Upon admission, median mRS score was 5 (IQR, 4–5). Intravenous thrombolysis was received by 58 (54%) of patients, and intraarterial thrombolysis was performed in 6 (6%) patients. Median initial ASPECTS was 10 (IQR, 8–10), and median final ASPECTS was 8 (IQR, 6–9). Median NIHSS at dismissal was 13 (IQR, 7–17). Median mRS score at dismissal was 4 (IQR, 4–5), and median mRS score 90 days post-stroke was 4 (IQR, 3–5).

mRS score 90 days post-stroke was better than mRS upon admission in 38 of 107 (36%) cases and better than or equal to mRS upon admission in 86 of 107 (80%) cases. Of the 93 patients with known pre-stroke mRS, 17 (18%) already had an mRS ≥ 3, with 15 of these 17 patients (88%) having a worse mRS score on admission than before. Of these 17 patients, 4 patients (24%) improved from admission, and 3 patients (18%) regained baseline.

### Treatment goal

**Fig. 2:**
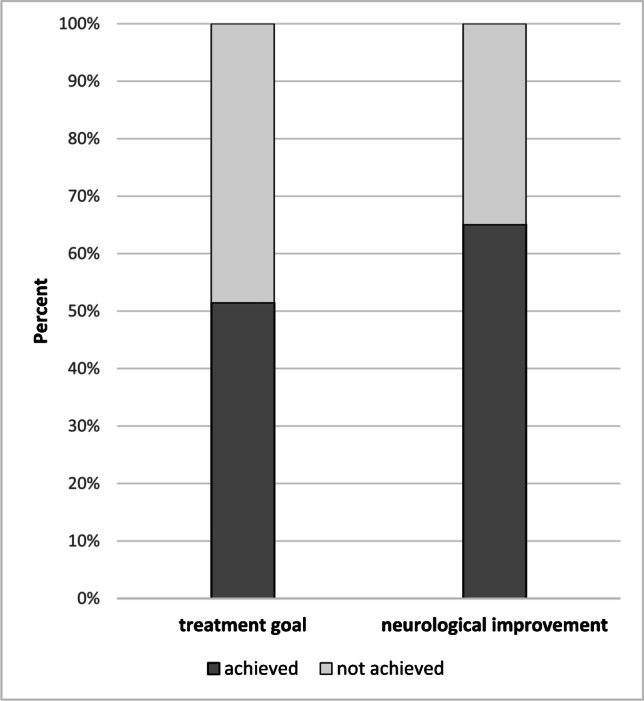
Achievement of treatment goal and neurological improvement

The treatment goal was achieved in 53 of 103 (52%) of patients (Fig. 2). Of these 53 patients, aphasia was prevented in 3 (6%) patients, paresis in 27 (51%) patients, a combination of aphasia and paresis in 18 (34%) patients, and a combination of neglect and paresis in 5 (9%) patients. Of the 50 patients, in whom we did not achieve the treatment goal, 14 (28%) improved neurologically, nevertheless.

Patient characteristics and outcome measures of patients, in whom we achieved our treatment goal, are summarized in Table 1. In summary, patients, in whom the preconceived treatment goal was achieved, had a significantly more favorable pre-stroke mRS score (*p* = 0.05), a lower mRS score upon admission (*p* = 0.001), and a better initial ASPECT score (*p* = 0.05). NIHSS upon admission was lower, without reaching statistical significance (*p* = 0.05). mRS score (*p* = 0.001) and NIHS score upon dismissal (*p* = 0.001), final ASPECT score (*p* = 0.001), and mRS score at 90 days (*p* = 0.001) were significantly better in the group, in whom the treatment goal was achieved. Other baseline and procedural characteristics such as number of thrombectomy passes and procedural times did not differ significantly between the two groups (*p* ≥ 0.15).

**Table 1:** Overview of baseline, procedural, and outcome characteristics in the groups treatment goal achieved vs. treatment goal not achieved (*n* = 103)

	Treatment goal achieved (n = 53)	Treatment goal not achieved (n = 50)	p value
Age [years] [median]	78 (IQR, 74–84)	77 (IQR, 69–82)	.188
Female sex [*n*]	36/53 (68%)	25/50 (50%)	.066
Occlusion site M1 occlusion/distal M1 included M2/carotid T occlusion [*n*]	31 (59%)/1 (2%)/21 (40%)	26 (52%)/3 (6%)/21 (42%)	.623
Stroke etiology: large-artery atherosclerosis/cardioembolic/undetermined and unknown/competing causes [*n*]	5 (9%)/43 (81%)/3 (6%)/2 (4%)	8 (16%)/34 (68%)/6 (12%)/2 (4%)	.979
Hypertension [*n*]	51/53 (96%)	45/50 (90%)	.212
Atrial fibrillation [*n*]	37/53 (70%)	27/50 (54%)	.100
Diabetes mellitus type II [*n*]	13/53 (25%)	17/50 (34%)	.293
Fat metabolism disorder [*n*]	21/53 (40%)	16/50 (32%)	.423
Adiposity [*n*]	24/53 (45%)	20/50 (40%)	.590
Nicotine abuse [*n*]	11/53 (21%)	9/50 (18%)	.725
Cardiovascular pre-existing illnesses [*n*]	39/53 (74%)	28/50 (56%)	.063
Previous stroke [*n*]	12/53 (23%)	10/50 (20%)	.745
mRS score pre-stroke [median]	0 (IQR, 0–0)	0 (IQR, 0–2)	.045*
mRS score upon admission [median]	4 (IQR, 4–5)	5 (IQR, 4–5)	.001*
NIHSS upon admission [median]	17 (IQR, 10–20)	19 (IQR, 16–21)	.054
Initial ASPECTS [median]	10 (IQR, 8–10)	9 (IQR, 8–10)	.047*
Intravenous thrombolysis [*n*]	33/53 (62%)	25/50 (50%)	.212
Intraarterial thrombolysis [*n*]	2/53 (4%)	4/50 (8%)	.362
Symptom-to-door [min] [median]	89 (IQR, 55–137)	85 (IQR, 46–125)	.626
Door-to-reperfusion [min] [median]	122 (IQR, 97–158)	135 (IQR, 105–190)	.153
Symptom-to-reperfusion [min] [median]	230 (IQR, 182–319)	229 (IQR, 195–348)	.629
eTICI pre-thrombectomy [median]	0 (IQR, 0–0)	0 (IQR, 0–0)	.610
Passes [median]	2 (IQR, 1–3)	2 (IQR, 1–3)	.518
Final ASPECTS [median]	8 (IQR, 7–10)	7 (IRQ, 4–8)	< .001*
mRS score at dismissal [median]	4 (IQR, 3–4)	5 (IQR, 4–5)	< .001*
NIHSS at dismissal [median]	7 (IQR, 3–12)	17 (IQR, 14–20)	< .001*
mRS score 90 days post-stroke [median]	4 (IQR, 3–5)	5 (IQR, 4–5)	< .001*
mRS score 90 days post-stroke better than or equal to mRS pre-stroke [*n*]	2/46 (4%)	0/44 (0%)	.164
mRS score 90 days post-stroke better than mRS score pre-stroke [*n*]	1/46 (2%)	0/44 (0%)	.328
mRS score 90 days post-stroke better than or equal to mRS score upon admission [*n*]	43/53 (81%)	43/50 (86%)	.508
mRS score 90 days post-stroke better than mRS score upon admission [*n*]	25/53 (47%)	13/50 (26%)	.027*

Percentages are rounded to the nearest whole number

*IQR* interquartile range, *mRS* modified Rankin scale, *NIHSS* National Institutes of Health Stroke Scale, *ASPECTS* Alberta Stroke Program Early CT Score, *eTICI* expanded Thrombolysis in Cerebral Infarction

^*^Significant values

Multivariable logistic regression analysis did not reveal factors (age, sex, NIHSS upon admission, and initial ASPECTS) independently associated with the achievement of the treatment goal. The graph can be found in the online supplemental data.

### Neurological improvement

Neurological improvement was achieved in 67 of 103 (65%) of patients (Fig. 2). Patient characteristics and outcome measures of patients, in whom we achieved neurologic improvement, are summarized in Table 2. In summary, patients, in whom neurological improvement was achieved, were older (*p* = 0.01) and had a better initial ASPECT score (*p* = 0.01). mRS score (*p* = 0.001) and NIHS score at dismissal (*p* = 0.001), final ASPECT score (*p* = 0.001), and mRS score at 90 days (*p* = 0.002) were significantly better in the group, in whom neurological improvement was achieved. Other baseline and procedural characteristics such as number of thrombectomy passes and procedural times did not differ significantly between the two groups (*p* ≥ 0.45).

**Table 2:** Overview of baseline, procedural, and outcome characteristics in the groups neurological improvement achieved vs. neurological improvement not achieved (*n* = 103)

	Neurological improvement achieved (n = 67)	Neurological improvement not achieved (n = 36)	p value
Age [years] [median]	79 (IQR, 76–84)	73 (IQR, 65–81)	.012*
Female sex [*n*]	43/67 (64%)	18/36 (50%)	.165
Occlusion site M1 occlusion/distal M1 included M2/carotid T occlusion [*n*]	40 (60%)/1 (2%)/26 (39%)	17 (47%)/3 (8%)/16 (44%)	.344
Stroke etiology: large-artery atherosclerosis/cardioembolic/undetermined and unknown/competing causes [*n*]	9 (13%)/52 (78%)/3 (5%)/3 (5%)	4 (11%)/25 (69%)/6 (17%)/1 (3%)	.247
Hypertension [*n*]	64/67 (96%)	32/36 (89%)	.204
Atrial fibrillation [*n*]	44/67 (66%)	20/36 (56%)	.315
Diabetes mellitus type II [*n*]	18/67 (27%)	12/36 (33%)	.493
Fat metabolism disorder [*n*]	25/67 (37%)	12/36 (33%)	.690
Adiposity [*n*]	27/67 (40%)	17/36 (47%)	.500
Nicotine abuse [*n*]	12/67 (18%)	8/36 (22%)	.600
Cardiovascular pre-existing illnesses [*n*]	45/67 (67%)	22/36 (61%)	.541
Previous stroke [*n*]	17/67 (25%)	5/36 (14%)	.177
mRS score pre-stroke [median]	0 (IQR, 0–1)	0 (IQR, 0–3)	.147
mRS score upon admission [median]	4 (IQR, 4–5)	5 (IQR, 4–5)	.058
NIHSS upon admission [median]	18 (IQR, 12–20)	18 (IQR, 15–21)	.407
Initial ASPECTS [median]	10 (IQR, 8–10)	9 (IQR, 7–10)	.012*
Intravenous thrombolysis [*n*]	42/67 (63%)	16/36 (44%)	.077
Intraarterial thrombolysis [*n*]	3/67 (5%)	3/36 (8%)	.428
Symptom-to-door [min] [median]	86 (IQR, 51–141)	94 (IQR, 50–121)	.857
Door-to-reperfusion [min] [median]	131 (IQR, 98–170)	127 (IQR, 104–164)	.599
Symptom-to-reperfusion [min] [median]	235 (IQR, 188–322)	214 (IQR, 192–390)	.948
eTICI pre-thrombectomy [median]	0 (IQR, 0–0)	0 (IQR, 0–0)	.972
Passes [median]	2 (IQR, 1–3)	2 (IQR, 1–3)	.445
Final ASPECTS [median]	8 (IQR, 7–10)	7 (IQR, 4–8)	< .001*
mRS score at dismissal [median]	4 (IQR, 3–4)	5 (IQR, 4–5)	< .001*
NIHSS at dismissal [median]	10 (IQR, 4–15)	18 (IQR, 15–21)	< .001*
mRS score 90 days post-stroke [median]	4 (IQR, 3–5)	5 (IQR, 4–5)	.002*
mRS score 90 days post-stroke better than or equal to mRS pre-stroke [*n*]	2/60 (3%)	0/30 (0%)	.315
mRS score 90 days post-stroke better than mRS score pre-stroke [*n*]	1/60 (2%)	0/30 (0%)	.480
mRS score 90 days post-stroke better than or equal to mRS score upon admission [*n*]	56/67 (84%)	30/36 (83%)	.974
mRS score 90 days post-stroke better than mRS score upon admission [*n*]	30/67 (45%)	8/36 (22%)	.024*

Percentages are rounded to the nearest whole number

*IQR* interquartile range, *mRS* modified Rankin scale, *NIHSS* National Institutes of Health Stroke Scale, *ASPECTS* Alberta Stroke Program Early CT Score, *eTICI* expanded Thrombolysis in Cerebral Infarction

^*^Significant values

Multivariable logistic regression analysis revealed that neurological improvement was independently associated with initial ASPECT score (OR, 1.41; CI, 1.09–1.82; *p* = 0.009), whereas age (OR, 1.02; CI, 0.99–1.06; *p* = 0.19), sex (OR, 1.67; CI, 0.7–3.98; *p* = 0.25), and NIHSS upon admission (OR, 0.98; CI, 0.9–1.05; *p* = 0.53) were not independently associated with neurological improvement. The graph is in the online supplemental data.

## Discussion

We analyzed our patients with good procedural outcome and formally unfavorable outcome of mRS 3–5 at 90 days and analyzed their clinical characteristics and the perceived unfavorable outcome. A major finding of our analysis is that even though all patients were formally classified as having unfavorable outcome, there was neurological improvement in approximately 65% of patients. However, such improvement is not necessarily treatment related but may simply reflect typical neurological improvement that is found in the majority of stroke patients during neurorehabilitation [24]. Nonetheless, our results imply that it is worth taking a closer look at neurological and clinical improvement: In fact, approximately 18% of patients (with known pre-stroke mRS) already had a poor mRS initially and thus could in theory not improve beyond this baseline status. Even though favorable outcome defined as functional independence could not be reached in these patients, mRS improved from admission or even reached the pre-stroke state in approximately 41% of these patients. Considering this improvement as a good outcome in this patient population, this result can be seen as a good outcome rate of 41% despite a formally unfavorable mRS of 3–5. When all patients are considered, mRS at 90 days compared with admission mRS improved in 36% of all patients and was stable or improved in 80% of all patients. In a real-life setting, the indication for thrombectomy is less restrictive in comparison to the criteria used in the clinical trials. Therefore, in patients with a pre-stroke functional dependence, with low ASPECT scores and with prolonged and unknown time windows until treatment who still received thrombectomy, the definition of treatment success must be less stringent. While in the early days of mechanical thrombectomy, such patients would not necessarily have been treated, the threshold has decreased over time, and even patients with poor baseline conditions are treated to avoid certain neurological deficits [25–27]. In what concerns treatment goals, we were able to achieve our goals in 52% of patients. Interestingly, we could not find independent predictors of achieving the treatment goal, whereas neurological improvement was achieved in patients with higher initial ASPECT scores. This can probably be explained by the fact that for specific neurological improvement, initial infarcted areas must be as small as possible, whereas for general disability, more complex factors come into play.

In summary, even though mRS has been shown to be both valid and reliable, our results argue for a more nuanced view of outcome, as actual benefit of treatment was more positive than the dichotomized mRS definition would suggest [17]. A more differentiated interpretation is more comprehensive, especially as dichotomization is not uniform in the literature. Because some studies regard a mRS of 0–2 as favorable, whereas others regard 0–1 as favorable, comparability is impaired [9, 10, 28, 29]. It should be stressed that mRS continues to be a robust and useful stroke outcome measure, but we argue that it should be interpreted in a more differentiated manner, especially when applied in real-life setting [30, 31]. This is supported by recent results of Ganesh et al. who showed that the ordinal assessment of mRS is superior to dichotomized assessment in real-life assessments of stroke outcome [32]. According to the authors, the ordinal mRS reflects patient disability best because it captures differences across the full range of the scale, and it may be misleading to consider certain conditions as equivalent [32]. Furthermore, the authors show that there are significant differences in long-term survival in patients with 90 days mRS scores of 3 to 5 with more favorable clinical courses in patients with lower mRS scores [32]. This is in line with our results that show that a therapeutic effort is worthwhile in a wider spectrum of patients and that not only patients with mRS ≤ 2 should be regarded as patients with favorable outcome. Alternative strategies for the evaluation of the benefit of acute treatment in ischemic stroke patients are the use of dichotomized mRS with stratification according to pre-stroke mRS with “return to baseline” as a favorable outcome criterion, the evaluation of the achievement of treatment goals (improvement in specific neurological deficits) established for each individual patient before beginning the acute treatment, the use of stroke-specific patient reported outcomes, [33] or the use of utility-weighted mRS, which is a measure that combines physician-reported outcome with patient-reported health-related quality of life. [34]

### Limitations

As our analysis is retrospective, it suffers from typical limitations of such a design, meaning that our results would profit from prospective validation. Most importantly, the retrospective identification of treatment goals for individual patients is subjective to a degree, and we cannot exclude a possible influence of other patient baseline characteristics, to which the two investigators were not blinded. To minimize this bias, the two investigators analyzed patient records and CT images independently. The exclusion of patients who achieved a successful but not complete reperfusion (TICI 2b and 2c) may have induced an overestimation of potential treatment benefits but allowed for the exclusion of potential bias induced by non-optimal reperfusion. Nonetheless, our results allow a more differentiated insight into this topic and may serve as a basis for future studies.

### Conclusions

In summary, our results imply that dichotomizing clinical outcome in favorable and unfavorable outcome limits the interpretation of treatment benefit after thrombectomy and may obscure the achievement of treatment goals in a real-life setting. In fact, mRS improved from baseline upon admission or even reached the pre-stroke state in approximately 41% of patients with pre-stroke functional dependence. Even though all patients were formally classified as having unfavorable 3-month outcome (mRS 3–5), there was neurological improvement in approximately two-thirds of patients, and our treatment goal was achieved in approximately half of patients.

## Availability of data

Data are available upon reasonable request.

## Supplementary Information

Below is the link to the electronic supplementary material.

Supplementary file1 (PDF 388 kb)

## Data Availability

There is no code.
