# Supplementary material for: Seeing the good in the bad: actual clinical outcome of thrombectomy stroke patients with formally unfavorable outcome
Source: Neuroradiology. 2022 Mar 8;64(7):1429–36. doi: 10.1007/s00234-022-02920-1 (PMC9177466; doi:10.1007/s00234-022-02920-1)
Supplement: Supplementary file 1 — Supplementary file1 (PDF 388 kb) [file 234_2022_2920_MOESM1_ESM.pdf]

Title: Seeing the good in the bad: actual clinical outcome of thrombectomy stroke patients with formally unfavorable outcome

Journal: Neuroradiology

Authors: Friederike Blum[1]; Charlotte Hager[1]; Homan Taufik[1]; Martin Wiesmann[1], MD; Dimah Hasan[1], MD; Arno Reich[2], MD; João Pinho[2], MD; Omid Nikoubashman[1], MD

Affiliations:

[1]: Department of Neuroradiology

[2]: Department of Neurology

University Hospital RWTH Aachen, Germany

Corresponding author:

Omid Nikoubashman

Neuroradiologie Universitätsklinikum Aachen

Pauwelsstr. 30

52074 Aachen, Germany

Phone: +492418089602

Email: onikoubashman@ukaachen.de

Online Fig. 1 Treatment goal. Odds ratios and 95% confidence intervals of a logistic regression analysis

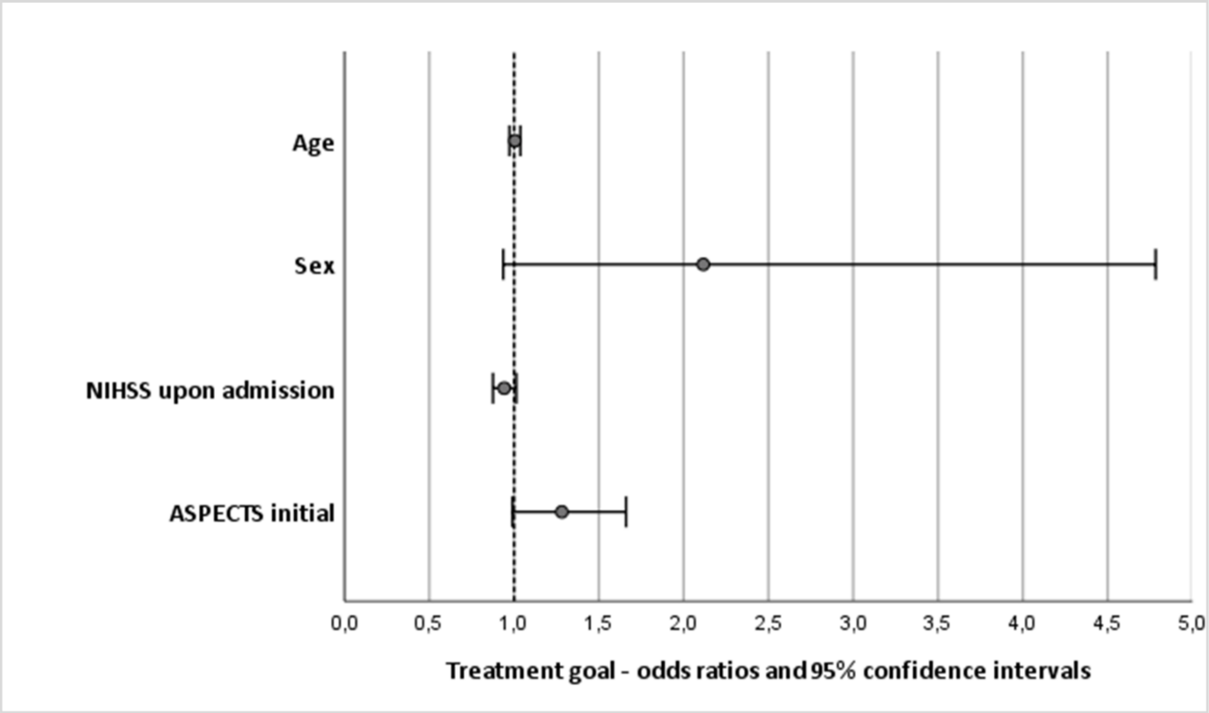

Online Fig. 2 Neurological improvement. Odds ratios and 95% confidence intervals of a logistic regression analysis

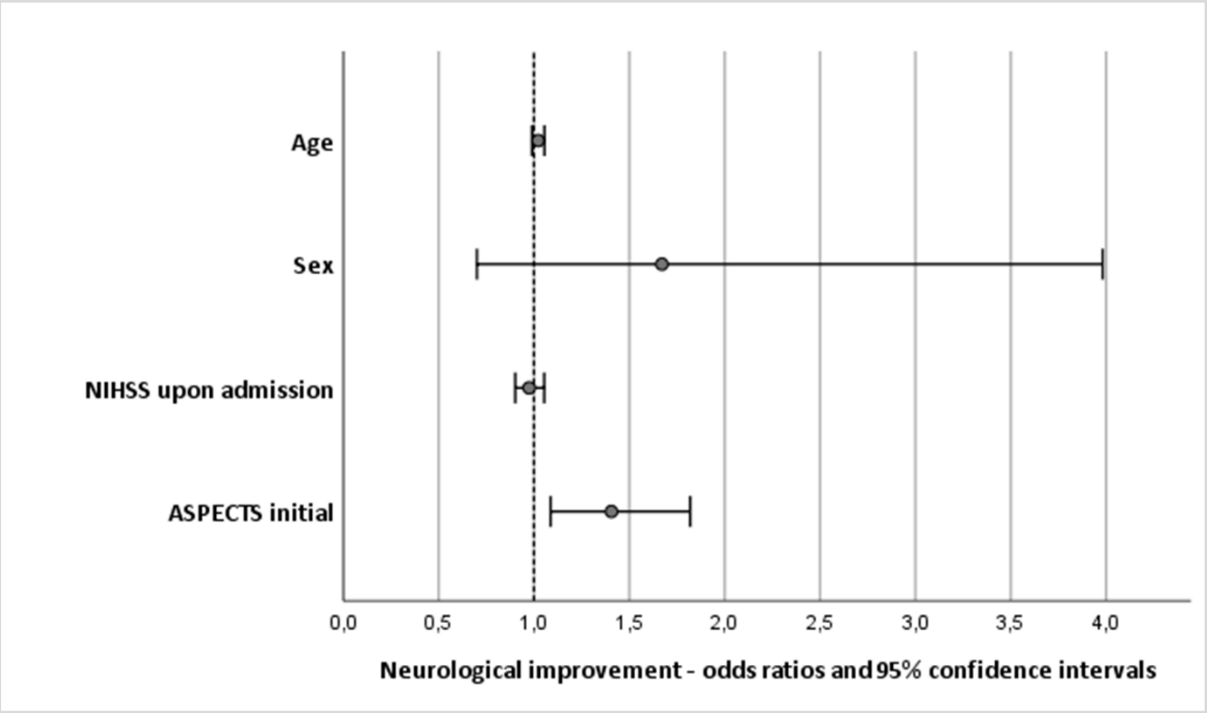

Online Table 1. Overview of Baseline, Procedural, and Outcome Characteristics in the total cohort (n=107).

	total cohort (n=107)
Age [years]][median]	77 (IQR, 71-83)
Female Sex [n]	64/107 (60%)
Occlusion site M1 occlusion / distal M1 included M2 / carotid T occlusion [n]	58 (54%) / 4 (4%) / 45 (42%)
Stroke etiology: large-artery atherosclerosis / cardioembolic / undetermined & unknown / competing causes [n]	13 (12%) / 81 (76%) / 9 (8%) / 4 (4%)
Hypertension [n]	99/106 (93%)
Atrial fibrillation [n]	66/106 (62%)
Diabetes mellitus type II [n]	31/106 (29%)
Fat metabolism disorder [n]	38/106 (36%)
Adiposity [n]	44/106 (41%)
Nicotine abuse [n]	20/106 (19%)
Cardiovascular pre-existing illnesses [n]	70/107 (65%)
Previous stroke [n]	22/106 (21%)
mRS score pre-stroke [median]	0 (IQR, 0-1)
mRS score upon admission [median]	5 (IQR, 4-5)
NIHSS upon admission [median]	18 (IQR, 12-20)
Initial ASPECTS [median]	10 (IQR, 8-10)
Intravenous thrombolysis [n]	58/107 (54%)
Intraarterial thrombolysis [n]	6/107 (6%)
Symptom-to-door [min] [median]	86 (IQR, 51-136)

Door-to-reperfusion [min] [median]	128 (IQR, 100-169)
Symptom-to-reperfusion [min] [median]	230 (IQR, 191-336)
eTICI pre-thrombectomy [median]	0 (IQR, 0-0)
Passes [median]	2 (IQR, 1-3)
Final ASPECTS [median]	8 (IQR, 6-9)
mRS score at dismissal [median]	4 (IQR, 4-5)
NIHSS at dismissal [median]	13 (IQR, 7-17)
mRS score 90 days post-stroke [median]	4 (IQR, 3-5)
mRS score 90 days post-stroke better than or equal to mRS pre-stroke [n]	3/93 (3%)
mRS score 90 days post-stroke better than mRS score pre-stroke [n]	1/93 (1%)
mRS score 90 days post-stroke better than or equal to mRS score upon admission [n]	86/107 (80%)
mRS score 90 days post-stroke better than mRS score upon admission [n]	38/107 (36%)

IQR indicates interquartile range; mRS, modified Rankin Scale; NIHSS, National Institutes of Health Stroke Scale; ASPECTS, Alberta Stroke Program Early CT Score; eTICI, expanded Thrombolysis in Cerebral Infarction.

Percentages are rounded to the nearest whole number.
